# Assessing anti-plaque efficiency of a herbal dentifrice

**DOI:** 10.6026/973206300213828

**Published:** 2025-10-31

**Authors:** Debadrita Ghosh, Vishal Yadav, Sonia Sharma, Nagaraja A, Nandini Komaravelli, Shali Singh

**Affiliations:** 1 Department of Pediatric & Preventive Dentistry, Consultant Pedodontist, Kolkata, West Bengal, India; 2 Department of Dentistry, Government Medical College, Bundi, Rajasthan, India; 3 Department of Dentistry, Maharaja Agrasen Medical College, Agroha, Haryana, India; 4 Department of Oral & Maxillofacial Pathology, Seema Dental College & Hospital, Rishikesh, Uttarakhand, India; 5 Department of Periodontology and Implantology, SVS Institute of Dental Sciences, Mahbubnagar, Telangana, India; 6 Department of Pedodontics & Preventive Dentistry, Safdarjung Hospital, New Delhi, India

**Keywords:** Herbal dentifrice, dental plaque, gingivitis, clinical trial, oral hygiene, *Azadirachta indica*, *Syzygium aromaticum*, *Melaleuca alternifolia*

## Abstract

Dental plaque is a key factor in gingivitis and periodontitis, requiring effective control measures. This randomized, double-blind clinical trial compared an herbal dentifrice (neem, clove and tea tree oil), a conventional fluoride dentifrice and a placebo in 90 participants with mild gingivitis. Plaque Index (PI) and Gingival Index (GI) were recorded at baseline, 2 weeks and 4 weeks. At 4 weeks, the herbal dentifrice showed the greatest reductions in PI (60.7%) and GI (55.7%) compared to fluoride (48.6%, 41.8%) and placebo (21.4%, 13.2%), with significant intergroup differences (p < 0.001). The herbal dentifrice demonstrated superior anti-plaque and anti-gingivitis efficacy, supporting its role as a natural oral hygiene alternative.

## Background:

Dental plaque, a structured biofilm adhering to tooth surfaces, is the primary etiological agent for gingivitis and periodontitis, affecting over 90% of the global population [1]. Mechanical plaque removal via toothbrushing with dentifrices remains the cornerstone of preventive oral care [2]. Conventional dentifrices typically contain synthetic antimicrobials (e.g., triclosan, stannous fluoride) or abrasives to disrupt plaque formation and maturation [3]. However, concerns regarding antimicrobial resistance, mucosal irritation and environmental persistence have driven interest in plant-based alternatives [4]. Herbal dentifrices leverage bioactive compounds with documented antimicrobial, anti-inflammatory and antioxidant properties, offering perceived safety and sustainability advantages [5]. Clinical evidence highlights significant advancements in oral hygiene technologies and formulations aimed at improving gingival and plaque control. Boneta *et al.* [6] demonstrated that a triclosan/copolymer and sodium fluoride dentifrice achieved superior reductions in supragingival plaque and gingivitis compared to stannous fluoride/zinc lactate toothpaste. Similarly, Nathoo *et al.* [7] reported that the use of an advanced sonic powered toothbrush with sensing and control technologies resulted in markedly greater improvements in plaque removal and gingival health than conventional manual brushing. Collectively, these studies emphasize the effectiveness of innovative oral care products in enhancing daily preventive dental care. Despite promising results, many studies suffer from methodological limitations, including small sample sizes, lack of blinding, short durations and inadequate control groups [8]. Furthermore, synergistic effects of multi-herbal formulations remain underexplored, particularly against established conventional benchmarks [9]. A critical research gap exists in rigorously evaluating the efficacy of novel multi-herbal dentifrices against both conventional and placebo controls over clinically relevant periods. Most trials focus on single-herb extracts, overlooking potential synergistic benefits of combined phytochemicals [10]. Additionally, few studies assess gingival health concurrently with plaque scores, limiting comprehensive efficacy conclusions [11]. This study addresses these gaps by evaluating a novel herbal dentifrice combining neem, clove and tea tree oil-ingredients with complementary antimicrobial and anti-inflammatory mechanisms [12, 13-14]. Therefore, it is of interest to determine the anti-plaque and anti-gingivitis efficacy of this herbal dentifrice compared to a conventional fluoride dentifrice and a placebo over a 4-week period.

## Materials and Methods:

## Study design:

A randomized, double-blind, controlled clinical trial with three parallel arms was conducted over 4 weeks. Participants were randomly assigned to Group A (herbal dentifrice), Group B (conventional fluoride dentifrice), or Group C (placebo dentifrice) using computer-generated block randomization (block size: 6). Allocation concealment was maintained via opaque sealed envelopes. Participants, examiners and data analysts were blinded to group assignments.

## Sample size:

Sample size was calculated using G*Power 3.1 (α = 0.05, power = 80%, effect size = 0.4). Based on prior PI reduction data [6], a minimum of 26 participants per group was required. Accounting for 15% attrition, 30 participants per group (total N = 90) were enrolled.

## Inclusion criteria:

[1] Adults aged 18-60 years.

[2] ≥ 20 natural teeth.

[3] Plaque Index (PI) score ≥ 1.5 and Gingival Index (GI) score ≥ 1.0 (mild gingivitis).

[4] Good general health (ASA I/II).

[5] Willingness to comply with the protocol.

## Exclusion criteria:

[1] Periodontitis (clinical attachment loss > 3 mm, probing depth > 4 mm).

[2] Systemic diseases (e.g., diabetes, immunosuppression).

[3] Antibiotic/anti-inflammatory use within 3 months.

[4] Pregnancy or lactation.

[5] Orthodontic appliances or extensive restorations.

[6] Known allergy to herbal ingredients or fluoride.

## Test materials:

[1] Group A (Herbal dentifrice): Base (calcium carbonate, glycerin, sodium lauryl sulfate) with 2% neem extract (*Azadirachta indica*), 0.5% clove oil (*Syzygium aromaticum*) and 0.3% tea tree oil (*Melaleuca alternifolia*). Standardized extracts were HPLC-verified for marker compounds (azadirachtin, eugenol, terpinen-4-ol).

[2] Group B (Conventional dentifrice): Commercial fluoride dentifrice (0.32% sodium fluoride, 1450 ppm fluoride).

[3] Group C (Placebo dentifrice): Base identical to Group A without active ingredients. All dentifrices were identical in color, flavor and packaging, coded by an independent pharmacist.

## Procedures:

[1] Baseline examination: PI (Silness & Löe, 1964) and GI (Löe & Silness, 1963) were recorded by two calibrated examiners (intraclass correlation coefficient > 0.85). Plaque was disclosed using erythrosine dye.

[2] Oral hygiene instruction: All participants received standardized brushing instruction (modified Bass technique) and a soft-bristled toothbrush.

[3] Intervention: Participants brushed twice daily (2 minutes) with assigned dentifrice. Compliance was monitored via brushing diaries and dentifrice weight checks.

[4] Follow-up examinations: PI and GI reassessed at 2 weeks and 4 weeks. Adverse events were recorded.

## Statistical analysis:

Data were analyzed using SPSS v26.0 (IBM, USA). Normality was assessed via Shapiro-Wilk test. Descriptive statistics (mean ± SD, percentages) were computed. Intergroup comparisons used one-way ANOVA with Tukey's post-hoc test. Intragroup changes were analyzed using paired t-tests. Percentage reductions were calculated as [(Baseline score - Final score)/Baseline score] x 100. p < 0.05 was considered significant.

## Results:

Of 102 screened individuals, 90 met criteria and were randomized. Five participants withdrew (Group A: 2, Group B: 1, Group C: 2; unrelated to adverse events), leaving 85 for analysis (Group A: 28, Group B: 29, Group C: 28). Baseline demographics and clinical parameters were comparable across groups (Table 1 - see PDF). All groups showed PI reductions at 4 weeks, but Group A demonstrated the greatest improvement (Table 2 - see PDF). At 4 weeks, Group A's PI reduction (60.7%) was significantly higher than Group B (48.6%, p < 0.001) and Group C (21.4%, p < 0.001). Intergroup differences were significant at both 2 weeks (p < 0.001) and 4 weeks (p < 0.001). Group A achieved superior GI reduction (55.7%) compared to Group B (41.8%, p < 0.001) and Group C (13.2%, p < 0.001) at 4 weeks (Table 3 - see PDF). Intergroup differences were significant at all-time points (p < 0.001). No adverse events were reported.

## Discussion:

This clinical trial demonstrated that a novel herbal dentifrice containing neem, clove and tea tree oil was significantly more effective in reducing plaque accumulation and gingival inflammation compared to a conventional fluoride dentifrice and a placebo. The herbal group achieved greater reductions in both plaque and gingival indices, with improvements exceeding 55-60% over 4 weeks, while the conventional group showed moderate reductions and the placebo group exhibited only minimal benefits. These results reinforce the potential of multi-herbal dentifrices as effective alternatives to conventional formulations for maintaining oral hygiene [6, 7]. The superior efficacy of the herbal dentifrice may be explained by the synergistic actions of its active constituents. Neem provides phytochemicals such as azadirachtin and nimbidin, which possess antibacterial activity and inhibit biofilm formation [12]. Clove oil contains eugenol, a compound with strong antimicrobial and anti-inflammatory properties, which reduces bacterial adhesion and gingival irritation [13]. Tea tree oil contributes terpinen-4-ol, known for its ability to penetrate biofilms and suppress anaerobic microorganisms associated with gingivitis [14]. Collectively, these compounds provide a multi-target antimicrobial and anti-inflammatory effect, contrasting with fluoride dentifrices, which primarily act through enamel remineralization and limited bacteriostatic effects [15]. The modest reductions observed in the placebo group are likely attributable to improved mechanical plaque removal as a result of enhanced oral hygiene awareness (Hawthorne effect) and adherence to standardized brushing instructions [16]. This highlights the importance of active antimicrobial and anti-inflammatory ingredients in dentifrices for achieving optimal plaque and gingivitis control. The study possesses several strengths, including its randomized, double-blind design, adequate sample size and use of validated clinical indices. However, some limitations should be acknowledged. The intervention period was relatively short (4 weeks), preventing assessment of long-term benefits. Additionally, microbiological and molecular analyses were not performed, which could have provided deeper insights into microbial shifts and biofilm dynamics. The trial also focused exclusively on participants with mild gingivitis; therefore, the applicability of findings to patients with moderate to severe periodontitis remains uncertain. Future studies should extend the observation period, incorporate microbial assessments and evaluate outcomes across different severities of periodontal disease.

## Conclusion:

A novel herbal dentifrice containing neem, clove and tea tree oil significantly reduces dental plaque and gingivitis compared to conventional fluoride and placebo dentifrices. The herbal group achieved a 60.7% reduction in Plaque Index and 55.7% reduction in Gingival Index over 4 weeks, outperforming both control groups. Data shows the potential of multi-herbal formulations as effective, natural alternatives for oral hygiene maintenance, addressing concerns about synthetic antimicrobials. Future research should explore long-term effects, mechanisms of action and efficacy in diverse populations.
